# Childhood Leukemia and 50 Hz Magnetic Fields: Findings from the Italian SETIL Case-Control Study

**DOI:** 10.3390/ijerph120202184

**Published:** 2015-02-16

**Authors:** Alberto Salvan, Alessandra Ranucci, Susanna Lagorio, Corrado Magnani

**Affiliations:** 1 Institute for Systems Analysis and Computer Science “Antonio Ruberti”, IASI-CNR, Via dei Taurini 19, 00185 Rome, Italy; E-Mail: alberto.salvan@gmail.com; 2 Medical Statistics & Cancer Epidemiology Unit—Department of Translational Medicine, CPO Piemonte and University of Eastern Piedmont, Via Solaroli 17, 28100 Novara, Italy; E-Mails: alessandra.ranucci@cpo.it (A.R.); corrado.magnani@cpo.it (C.M.); 3 National Centre for Epidemiology, Surveillance and Health Promotion—National Institute of Health, Viale Regina Elena 299, 00161 Rome, Italy

**Keywords:** leukemia, acute lymphoblastic leukemia, childhood, extremely low frequency (ELF) magnetic fields, epidemiology, case-control study

## Abstract

We report on an Italian case-control study on childhood leukemia and exposure to extremely low frequency magnetic fields (ELF-MF). Eligible for inclusion were 745 leukemia cases, aged 0–10 years at diagnosis in 1998–2001, and 1475 sex- and age-matched population controls. Parents of 683 cases and 1044 controls (92% *vs.* 71%) were interviewed. ELF-MF measurements (24–48 h), in the child’s bedroom of the dwelling inhabited one year before diagnosis, were available for 412 cases and 587 controls included in the main conditional regression analyses. The magnetic field induction was 0.04 μT on average (geometric mean), with 0.6% of cases and 1.6% of controls exposed to >0.3 μT. The impact of changes in the statistical model, exposure metric, and data-set restriction criteria was explored via sensitivity analyses. No exposure-disease association was observed in analyses based on continuous exposure, while analyses based on categorical variables were characterized by incoherent exposure-outcome relationships. In conclusion, our results may be affected by several sources of bias and they are noninformative at exposure levels >0.3 μT. Nonetheless, the study may contribute to future meta- or pooled analyses. Furthermore, exposure levels among population controls are useful to estimate attributable risk.

## 1. Introduction

Extremely low frequency magnetic fields (ELF-MF) were classified by the International Agency for Research on Cancer (IARC) as possibly carcinogenic (group 2B), based on limited evidence in humans, inadequate experimental support, and lack of plausible mechanisms at the exposure levels observed in epidemiological studies [1]. Such judgment was endorsed by a subsequent weight of evidence assessment carried out by the World Health Organization (WHO) [2].

The IARC evaluation was driven by a two-fold increase in risk of childhood leukemia (CL) among the exposed above 0.3–0.4 μT observed in two partially overlapping pooled analyses of studies published up to 1999 [3,4]. A pooled analysis of seven studies published later on (up to 2010) broadly replicated the earlier findings [5]. Recent critical reviews of the evidence concluded that there is still limited evidence for an association between CL and ELF-MF; the association is consistent and apparently specific, but its causality is still questionable [6,7].

It has been suggested that without further improvements in exposure assessment, control of bias and confounding, and knowledge of biological mechanisms, epidemiological studies will not be capable to contribute further insights on the topic [8,9].

That notwithstanding, nationwide measurement-based case-control studies of childhood leukemia and ELF-MF have the merit of providing information on exposure levels in the target populations, thus contributing to the public health impact assessment at the national and international levels. We report on findings from an epidemiological study (SETIL) with the above mentioned features.

## 2. Methods

### 2.1. Study Design

The SETIL case-control study is a collaborative project concurrently carried out by 15 research teams in 14 Italian regions (Piedmont, Liguria, Lombardy, Veneto, Friuli-Venezia-Giulia, Emilia-Romagna, Tuscany, Umbria, Marche, Latium, Campania, Puglia, East Sicily, West Sicily, Sardinia), according to a common protocol.

All incident cases of acute lymphoblastic (ALL) or acute non-lymphoblastic (AnLL) leukaemia in children aged 0–10 years at diagnosis in 1998–2001, and resident in the participating regions, were eligible for enrolment. The target population corresponds to 78% of Italian children in the selected age range (87% in Northern Italy, 100% in Central Italy, 61% in Southern Italy). The nationwide database of pediatric cancer, run by the Italian Association of Pediatric Hematology and Oncology (AIEOP), was used as case-ascertainment source. The estimated coverage of the AIEOP database for childhood leukemia (all types) was 97% over the period 1989–2006 [10,11]. In the Latium region only, a supplementary ad hoc search of eligible cases was carried out through the hospital discharge files of the main Rome pediatric hospital (not included at the time in the AIEOP network).

Two controls per case, matched on sex, date of birth (±15 days) and province of residence, were randomly selected from the Local Health Authority rolls of each participating region. These rolls are virtually complete, and regularly updated, lists of the resident population in each region. Non-participant controls were not replaced. The date of diagnosis was available from the AIEOP database; a reference date was assigned to each control, equal to the date of diagnosis of his/her matched case.

### 2.2. Ethical Approval

The SETIL study was approved by the Ethical Review Board of the Piedmont Region (authorization n. 2886, 15 February 1999), and by the relevant board of each research centre.

### 2.3. Approach and Interview

Parents of cases were first approached by the attending oncologists, usually after the induction phase of treatment. Contact of families of children deceased before the study start date was delayed at discretion of the attending physician.

Controls’ general practitioners were informed by mail about the child’s enrolment in the study and were asked to report objections, if any.

Requests for participation were sent by mail to the families of eligible children with medical approval, followed by phone calls to arrange an interview at home with both parents. Information regarding the parent not participating in the interview was eventually provided by the attending spouse and confirmed or elicited on the phone.

The questionnaire used in the interview included questions on parental educational level; parental occupational history; reproductive history of the mother, duration of the index pregnancy, child’s conditions at birth (birth weight and possible congenital birth defects, including Down syndrome), breast feeding; medical history of the child (X-rays, childhood diseases and immunizations); lifelong residential history of the child and the mother (during pregnancy), with details on all dwellings including full address, location (urban or rural), nearby traffic density, and proximity to power lines and broadcasting stations; child’s exposure to chemicals at home (with focus on solvents, pesticides, and second-hand tobacco smoke); maternal (during pregnancy) and child’s exposure to electrical appliances at home; school history of the child, including age at first attendance and class size. Further details on the study design, methods and descriptive results are provided elsewhere [12].

### 2.4. ELF-MF Measurements

Parents of children still living in the home inhabited one year before the date of diagnosis (or reference date for controls) were invited to participate in an ELF-MF measurement survey, pre-tested in a pilot study [13].

The indoor level of magnetic field induction was measured by portable meters. Long-term measurements (48 h following the protocol) were made in the child’s bedroom, using EMDEX^®^ meters (Enertech Consultants, Campbell, CA, USA) , models II or Lite, with sampling interval set to 30 s, placed under or close to the bed. The meters were encased in sealed plastic boxes to avoid tampering, and parents were instructed not to move them from the chosen location.

The detection limit (DL) of the EMDEX meters is 0.01 μT [14,15]. All meters were calibrated at 50 Hz every 6 months, as well as whenever suspicious results were observed. All sets of measurements were downloaded and inspected graphically using the EMCALC^©^ software (Enertech Consultants, Campbell, CA, USA); defective sets of measurements were excluded and, whenever possible, repeated.

### 2.5. Exposure Variables

ELF-MF bedroom measurements were summarized according to different metrics (arithmetic mean, geometric mean, 90th, 95th, and 99th percentiles). To calculate the geometric mean, instantaneous values below the DL were replaced with a very small non-null value (0.0001 μT). In order to explore the sensitivity of findings to the treatment of non-detects, corrected values of all individual summary metrics (arithmetic mean, geometric mean, and percentiles) were also calculated by replacing all null instantaneous measurements with two functions of the DL, namely ½ DL (0.005 μT), or DL/√2 (0.0071 μT).

### 2.6. Statistical Analyses

The main analyses of the relation between ELF-MF exposure and risk of childhood leukemia were based on conditional logistic regression for matched sets (CLR, Clogit procedure in STATA v. 11) [16]. Candidate to inclusion were all participating cases and controls fulfilling the following protocol requirements: (a) the difference between birth dates of the case and his/her matched control(s) was not larger than ±15 days; (b) the house where the ELF-MF bedroom measurements were made corresponded to the home inhabited by the child one year before diagnosis/reference date; (c) the measurement duration was ≥24 h.

We checked the sensitivity of results to changes in the: (i) exposure metric, (ii) statistical model, (iii) leukemia cell lineage; (iv) dataset composition. To the first aim, odds ratios (ORs) and 95% confidence intervals (95% CI) were calculated in relation to three different exposure metrics (arithmetic mean, geometric mean, and 95th percentile). Moreover, since it is reasonable to assume that overnight values of ELF-MF bedroom measurements are more accurate exposure proxies than 24–48 h measurements, and might also have greater biologic relevance, a second series of CLR analyses were carried out using nighttime (10 pm to 5:59 am) recorded values as exposure variables.

Each exposure metric was included in a (separate) CLR model either as a continuous variable (arithmetic mean only), or as a categorical variable in three levels (≤0.1; (0.1–0.2]; >0.2 μT). Based on such a categorization, the great majority of our study subjects are classified as “non-exposed” (reference category ≤0.1 μT). A four-level categorization (≤0.1 μT; (0.1–0.2 μT]; (0.2–0.3 μT]; >0.3 μT) was only attainable when using the 95th percentile. The cutpoints were chosen for consistency with previous studies on the same topic.

Sensitivity analyses were also performed to assess variations possibly associated with different methods of correction of instantaneous values below the detection limit.

Possible variations in findings due to the statistical model (point ii. above) were explored in analyses based on unconditional regression models (ULR, Logistic procedure in STATA v. 11), adjusting for the matching variables: Sex, age at diagnosis (in four classes: [0,2), [2,4), [4,6), [6,10]), and area of residence (four categories: Lombardy, North excluding Lombardy, Centre, South and Islands).

To the third aim (possible changes due to leukemia type), all previously described analyses were repeated on a dataset restricted to ALL cases and their matched controls. Due to numerical constraints, it was not possible to assess the effect of ELF-MF exposure on risk of acute myeloid leukemia or other rarer types of AnLL.

To the fourth purpose (possible changes due to variation in the dataset composition), ULR analyses were performed on two different datasets: A first one consisting of cases and controls, matched and unmatched, satisfying all three criteria for inclusion in the CLR analyses (URL-1 models); a second larger dataset including all participating cases and controls satisfying criteria “b” and “c” above, independent of the age-matching criterion (URL-2 models).

Additional sensitivity analyses (based on ULR-2 models, and all leukemias) were carried out on datasets further restricted to: (a) children without Down syndrome (6 cases excluded); (b) subjects residing since conception and/or birth in the home inhabited one year before diagnosis (where the ELF-MF measurements were made); (c) measurements with duration ≥48 h; (d) measurements with sampling intervals of 30 s; (e) measurements made during the week-end.

The covariates considered as potential confounding variables included: Subject’s characteristics (birth weight, breast feeding, birth order, number of siblings, exposure to second-hand smoke before diagnosis or corresponding reference date for controls); parental traits (maternal and paternal age at the index birth, maternal and paternal educational level, maternal smoking during the index pregnancy, maternal exposure to second-hand smoke during the index pregnancy; maternal smoking at interview); features of the home inhabited at the time of the ELF-MF measurements (detached house *vs.* apartment in multi-level building; square meters per tenant; self-reported traffic density in the home proximity); variables related to measurement setting such as type of exposure meter (EMDEX II *vs.* EMDEX Lite), season, delay between diagnosis/reference date and interview, delay between interview and ELF-MF measurements.

The selection of actual confounders, to adjust for in the analyses (CLR or ULR), was not algorithm-driven. Potential confounding variables were added, one at a time, to each logistic model including the ELF-MF exposure metric only; the performance of each confounder-including model was then compared with that of the baseline model using the likelihood ratio test for nested models (CLR analyses), or by the AIC and BIC statistics (ULR analyses).

The limited sample size in the upper categories of the exposure metrics precluded the assessment of possible interactions between the exposure metrics and potential effect-modifiers.

## 3. Results

### 3.1. Participation

**Figure 1:**
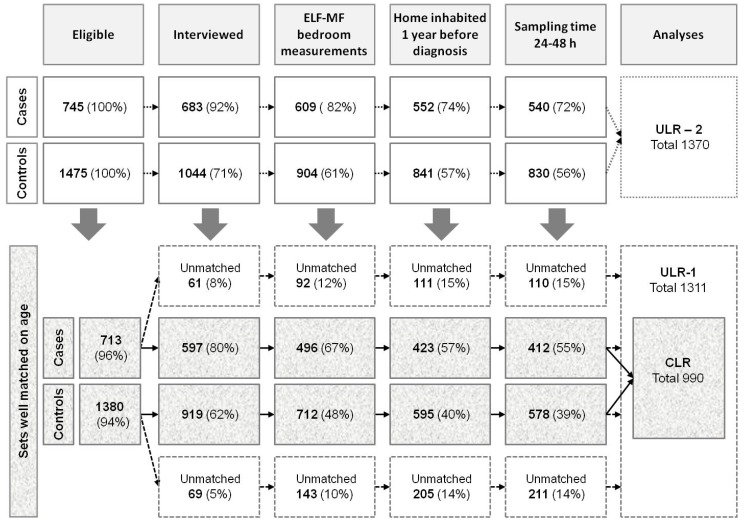
Participation rates and inclusion in the datasets for the analyses. CLR = dataset of cases and controls strictly matched on age (difference in dates of birth not larger than ±15 days), with valid measurements, included in the main analyses based on conditional logistic regression models; ULR-1 = dataset of matched and unmatched cases and controls with valid measurements from sets originally strictly matched on age, included in a first series of unconditional logistic regression models; ULR-2 = dataset of matched and unmatched cases (with valid measurements but independent of compliance with the strict age-matching criterion) included in a second series of unconditional logistic regression models.

Eligible for inclusion were 745 cases (647 ALL; 98 AnLL) and 1475 sex- and age-matched controls. The envisaged 1:2 case-control ratio proved attainable for 736 cases (99%) who were matched to 1472 controls; three cases were matched to one control each; no suitable control could be found for six cases. The age-matching criterion (difference in dates of birth not larger than ± 15 days) was relaxed in a few instances (26 cases and 95 controls), so that the data-set candidate to the main analyses (CLR), or to the ULR-1 analyses, was reduced to 713 cases (619 ALL; 94 AnLL) and 1380 controls (Figure 1).

Participation rates were higher among cases compared to controls. Overall, 92% of case-parents were interviewed *vs.* 71% of controls. The proportion of eligible children participating in the ELF-MF measurement protocol was 82% among cases *vs.* 61% among controls; however, measurements fully complying with the protocol were available for 72% of cases, and 56% of controls, candidate to the ULR-2 analyses (Figure 1).

The case-control differential in participation rates (≈20%) did not increase as far as the adherence burden grew, moving from acceptance of the interview to availability of valid long-term ELF-MF bedroom measurements (Figure 1). Due to the restrictions applied, only 55% and 39% of eligible cases and controls qualified for inclusion in the CLR analyses, and the corresponding proportions in the ULR-1 analyses are 70% *vs.* 53% (Figure 1).

The main reasons for non-participation, among non-interviewed subjects (62 cases, 431 controls), were parents’ refusal (26 cases, 303 controls; 42% and 70% of non-interviewed cases and controls, respectively), inability to trace (six cases, 80 controls; 10% *vs.* 19%), lack of consent by the attending physician to contact families (seven cases, 27 controls; 11% *vs.* 6%), and death of the child (21 cases, no control; 34% *vs.* 0%); the families of 14 controls (3% of non-interviewed) matched to non-participating cases were not approached; the research team decided not to interview parents of adopted children (two cases, and three controls) or caregivers of one control child in orphanage since birth; the reason of non-participation was unknown for three controls.

### 3.2. Delay Diagnosis-Interview and Interview-Measurements

The families of participating cases were interviewed on average 15 months (SD 6.5) after the date of diagnosis, and the control families 18 months (SD 7.9) after the corresponding reference date. The delay between interview and ELF-MF measurements was 1.5 months (SD 5.4) among cases, and 1.7 months (SD 6.5) among controls.

### 3.3. Personal Characteristics

Cases and controls were comparable in terms of gender and age, while control-parents (both father and mother) were more educated than case-parents (Table 1). Cases, compared to controls, were a little more often single children or had fewer siblings. There were modest differences between cases and controls in the proportions of mothers smoking during pregnancy, and of children exposed to second-hand smoke at diagnosis (both slightly higher among cases than controls). At the time of interview and measurements, the prevalence of children living in detached houses, as opposed to apartment building, was slightly higher among controls than cases.

**Table 1:** Descriptive characteristics of cases and controls.

	Cases						Controls					
	Eligible		Interviewed		Matched		Eligible		Interviewed		Matched	
	N°	(%)	N°	(%)	N°	(%)	N°	(%)	N°	(%)	N°	(%)
**Sex**												
Male	406	(54.5)	370	(54.2)	224	(54.4)	797	(54.0)	562	(53.8)	309	(53.5)
Female	339	(45.5)	313	(45.8)	188	(45.6)	678	(46.0)	482	(46.2)	269	(46.5)
**Age at Diagnosis (Years)**												
[0,2)	108	(14.5)	95	(13.9)	59	(14.3)	210	(14.2)	156	(14.9)	83	(14.4)
[2,4)	255	(34.2)	243	(35.6)	144	(35.0)	493	(33.4)	351	(33.6)	201	(34.8)
[4,6)	162	(21.7)	146	(21.4)	87	(21.1)	322	(21.8)	233	(22.3)	120	(20.8)
[6,10]	220	(29.5)	199	(29.1)	122	(29.6)	427	(28.9)	304	(29.1)	174	(30.1)
**Father’s Education**												
Primary school (8 years)	-	-	340	(49.8)	205	(49.8)	-	-	463	(44.3)	246	(42.5)
High school (12–13 years)	-	-	268	(39.2)	161	(39.1)	-	-	424	(40.6)	239	(41.3)
University (≥15 years)	-	-	70	(10.3)	43	(10.4)	-	-	151	(14.5)	88	(15.2)
Missing	-	-	5	(0.73)	3	(0.73)	-	-	6	(0.57)	5	(0.87)
**Mother’s Education**												
Primary school (8 years)	-	-	320	(46.9)	188	(45.6)	-	-	400	(38.3)	207	(35.8)
High school (12–13 years)	-	-	285	(41.7)	179	(43.5)	-	-	503	(48.2)	286	(49.5)
University (≥15 years)	-	-	78	(11.4)	45	(10.9)	-	-	139	(13.3)	85	(14.7)
Missing	-	-	0	(-)	0	(-)	-	-	2	(0.19)	0	(-)
**Children in the Family**												
1	-	-	192	(28.1)	114	(27.7)	-	-	290	(27.8)	141	(24.4)
2	-	-	364	(53.3)	220	(53.4)	-	-	565	(54.1)	323	(55.9)
3+	-	-	127	(18.6)	78	(18.9)	-	-	189	(18.1)	114	(19.7)
**Mother’s Smoking (during Pregnancy)**												
Yes	-	-	83	(12.2)	50	(12.1)	-	-	115	(11.0)	61	(10.5)
No	-	-	599	(87.7)	362	(87.9)	-	-	927	(88.8)	516	(89.3)
Missing	-	-	1	(0.15)	0	(-)	-	-	2	(0.19)	1	(0.17)
**Child’s Exposure to Second-Hand Smoke (at Diagnosis)**												
Yes	-	-	221	(32.4)	134	(32.5)	-	-	312	(29.9)	176	(30.4)
No	-	-	460	(67.3)	276	(67.0)	-	-	724	(69.3)	397	(68.7)
Missing	-	-	2	(0.29)	2	(0.49)	-	-	8	(0.77)	5	(0.87)
**Type of Home (at Interview and Measurements)**												
Detached house	-	-	167	(24.4)	100	(24.3)	-	-	260	(24.9)	154	(26.6)
Apartment building	-	-	503	(73.7)	305	(74.0)	-	-	749	(71.7)	419	(72.5)
Missing	-	-	13	(1.9)	7	(1.7)	-	-	35	(3.4)	12	(0.87)

Matched = case-control sets strictly matched on age (difference in dates of birth not larger than ±15 days), with valid ELF-MF measurements (made in the home inhabited one year before diagnosis/reference date, and duration ≥24 h), included in the main CLR analyses.

The distribution of interviewed cases and controls by the selected characteristics considered herein was not substantially altered by the restrictions applied to inclusion in the matched analyses (Table 1).

This also applies to the subsets of children included in the ULR-1 and ULR-2 analyses, as shown in the corresponding table available as online Supplementary Material (Table S1).

### 3.4. Estimated Exposure to ELF-MF

Long-term measurements of 50 Hz magnetic field were performed in the bedroom of 609 cases and 904 controls. Not all available measurements, however, complied with the protocol requirements. The subset of subjects with measurements ineligible for the analyses included 57 cases and 63 controls living at the time of the survey in homes different from those inhabited one year before the diagnosis/reference date, 12 cases and 11 controls with measurement duration <24 h, and one control meeting both exclusion criteria. Thus, the dataset examined in the current section consists of 1370 children (540 cases and 830 controls) with valid measurements, candidate to the ULR-2 analyses (Figure 1).

The average measurement duration in this dataset was 62 h (SD 22.6 h; range 24.1 to 181.9 h); measurement duration was between 24–48 h for 20% cases and 21% controls. The relative proportions of cases and controls with bedroom measurements ≥48 h was quite stable across analytical dataset (81% cases *vs.* 80% controls in the ULR analyses; 81% of both cases and controls in the CLR analyses).

Exposure meters were set to a sampling interval of 30 s, with a few exceptions (4 s = nine cases and five controls; 60 s = one control).

More cases than controls had bedroom measurements made during the week-end (45% *vs.* 40%, 57% *vs.* 55%, and 58% *vs.* 54% in the CLR, ULR-1, and ULR-2 datasets, respectively). Our best estimate of the intensity and variability of ELF-MF exposure in our target population, based on findings from all controls with valid measurements, is outlined in Table 2.

**Table 2:** ELF-MF level (μT) in the child’s bedroom among controls with valid measurements.

	Entire Sampling Time(# 830)				Overnight Sampling (# 830)				Subsample Week-End (# 452)			
Metric	Mean	SD	Min	Max	Mean	SD	Min	Max	Mean	SD	Min	Max
**AM**	0.045	0.121	0	2.52	0.043	0.133	0	2.509	0.042	0.133	0	2.55
**GM**	0.038	0.111	0.0001	2.50	0.040	0.132	0.0001	2.507	0.035	0.130	0.0001	2.51
**P90**	0.069	0.156	0	2.73	0.059	0.149	0	2.620	0.064	0.160	0	2.79
**P95**	0.079	0.165	0	2.81	0.065	0.154	0	2.650	0.076	0.170	0	2.83
**P99**	0.110	0.208	0	2.88	0.079	0.166	0	2.670	0.104	0.200	0	2.89

Valid measurements = made in homes inhabited one year before reference date and duration ≥24h; Overnight sampling = from 10 pm to 5:59 am; AM = arithmetic mean of instantaneous values from 24 to 48 h bedroom measurements; GM = geometric mean of instantaneous values from 24 to 48 h bedroom measurements; P90, P95, P99 = 90th, 95th and 99th percentiles of instantaneous values from 24 to 48 h bedroom measurements.

Corrections of non-detects (made at the level of individual instantaneous recordings) resulted in variable amount of change in average exposure levels by group, depending on the value assigned to measurements below the detection limit (0.0001, 0.005, or 0.007), and the metric (the geometric mean being more sensitive than the arithmetic mean, whereas the 90th, 95th, and 99th percentiles were almost unaffected) (Supplementary Table S2).

The proportion of subjects in the upper exposure category (>0.3 μT) varied across exposure metrics, ranging between 0.6% for the geometric mean (GM) to 8% for the 99th percentile (P99) among cases, and between 1.6% (GM) and 7% (P99) among controls (Table 3).

**Table 3:** Distribution of cases and controls with valid measurements by categories of exposure metrics.

Exposure Metric		Exposure Category							
		≤0.1 μT		(0.1–0.2] μT		(0.2–0.3] μT		>0.3 μT	
		Cases	Controls	Cases	Controls	Cases	Controls	Cases	Controls
**AM**	N°	485	758	41	43	9	12	5	17
	(%)	(89.81)	(91.33)	(7.59)	(5.18)	(1.67)	(1.45)	(0.93)	(2.05)
Mean AM (μT)		0.022	0.024	0.143	0.135	0.227	0.236	0.604	0.633
**GM**	N°	497	770	35	39	5	8	3	13
	(%)	(92.04)	(92.77)	(6.48)	(4.70)	(0.93)	(0.96)	(0.56)	(1.57)
Mean GM (μT)		0.019	0.020	0.145	0.137	0.238	0.248	0.741	0.638
**P90**	N°	456	694	50	82	19	18	15	36
	(%)	(84.44)	(83.61)	(9.26)	(9.88)	(3.52)	(2.17)	(2.78)	(4.34)
Mean P90 (μT)		0.028	0.028	0.154	0.148	0.245	0.250	0.566	0.592
**P95**	N°	438	663	54	102	28	25	20	40
	(%)	(81.11)	(79.88)	(10.00)	(12.29)	(5.19)	(3.01)	(3.70)	(4.82)
Mean P95 (μT)		0.030	0.030	0.152	0.148	0.251	0.248	0.579	0.606
**P99**	N°	400	605	65	115	30	49	45	61
	(%)	(74.07)	(72.89)	(12.04)	(13.86)	(5.56)	(5.90)	(8.33)	(7.35)
Mean P99 (μT)		0.036	0.036	0.150	0.150	0.246	0.246	0.718	0.662

Valid measurements = made in homes inhabited one year before reference date, and duration ≥24 h; AM = arithmetic mean of instantaneous values from 24 to 48 h bedroom measurements; GM = geometric mean of instantaneous values from 24 to 48 h bedroom measurements; P90, P95, P99 = 90th, 95th and 99th percentiles of instantaneous values from 24 to 48 h bedroom measurements.

### 3.5. Exposure-Outcome Relationship

Of the many potential confounding factors assessed (see the Methods section), only the educational level attained by father and mother proved to be consistently associated with the outcome of interest, and able to modify the measures of association between childhood leukemia and ELF-MF exposure, although the change was never greater than 10%.

The main findings from the analyses based on CLR models for matched sets, adjusting for parental education, are summarized in Table 4 with reference to all leukemias (left), or to ALL only (right).

There was no association between childhood leukemia risk and ELF-MF exposure estimates based on the time weighted average (TWA) of long-term bedroom measurements as a continuous variable (arithmetic mean), independent of disease morphology (all leukemias *vs.* ALL only).

When categorical average exposure variables were used, the risk of disease was apparently increased in the low-exposure class ((0.1–0.2] μT), and decreased at the upper exposure level (>0.2 μT), compared to the reference category (<0.1 μT). For example, according to the exposure classification based on the arithmetic mean of ELF-MF bedroom measurements, the ORs for all leukemias were 1.87 (1.04–3.34) for the category (0.1–0.2] μT, and 0.79 (0.35–1.79) at >0.2 μT; when the geometric mean was used, the ORs were 1.72 (0.95–3.13) at (0.1–0.2] μT, and 0.42 (0.13–1.37) at >0.2 μT (Table 4). Notably, in both instances, the confidence interval of the risk estimate for the higher exposure category did not include the point estimate of effect at the lower-exposure level.

**Table 4:** ORs for childhood leukemia according to ELF-MF exposure (CLR—Adjusted *).

		All Leukemias				Acute Lymphocytic Leukemia			
Exposure Metric	Level (μT)	Cases	Controls	OR	95% CI	Cases	Controls	OR	95% CI
AM continuous	per 1 μT increase	409	569	0.89	0.19–4.20	356	499	1.13	0.21–5.96
AM categorical	≤0.1	369	528	1.00	-	322	464	1.00	-
	(0.1–0.2]	30	24	1.87	1.04–3.34	24	19	1.77	0.94–3.33
	>0.2	10	17	0.79	0.35–1.79	10	16	0.88	0.38–2.00
GM categorical	≤0.1	378	534	1.00	-	330	468	1.00	-
	(0.1–0.2]	27	24	1.72	0.95–3.13	22	21	1.49	0.80–2.80
	>0.2	4	11	0.42	0.13–1.37	4	10	0.49	0.15–1.63
P95 (3 levels)	≤0.1	335	463	1.00	-	293	408	1.00	-
	(0.1–0.2]	39	69	0.80	0.52–1.22	33	60	0.76	0.47–1.21
	>0.2	35	37	1.22	0.73–2.02	30	31	1.26	0.73–2.15
P95 (4 levels)	≤0.1	335	463	1.00	-				
	(0.1–0.2)	39	69	0.81	0.53–1.25				
	(0.2–0.3)	20	13	2.24	1.03–4.88				
	>0.3	15	24	0.75	0.38–1.50				

* CLR adjusted = conditional logistic regression models, adjusted for parents’ education; AM = arithmetic mean of instantaneous values from 24 to 48 h bedroom measurements; GM = geometric mean of instantaneous values from 24 to 48 h bedroom measurements; P95 = 95th percentile of instantaneous values from 24 to 48 h bedroom measurements.

A different pattern appeared to emerge when the exposure classification was based on the 95th percentile (P95) of instantaneous recordings in individual bedroom measurements, in three level categories; e.g., the ORs for all leukemias were 0.80 (0.52–1.22) at (0.1–0.2] μT, and 1.22 (0.73–2.02) at >0.2 μT (Table 4). However, when a 4-level categorization of P95 was adopted (made feasible by the larger number of exposed subjects resulting from the use of this metric), the risk estimates across exposure categories returned to the puzzling pattern observed with the average exposure metrics; e.g., the ORs for all leukemias at (0.1–2] μT, (0.2–0.3] μT, and >0.3 μT were 0.81 (0.53–1.25), 2.24 (1.03–4.88), and 0.75 (0.38–1.50), respectively (Table 4).

Broadly similar results were obtained in the CLR analyses restricted to ALL cases and their matched controls, notwithstanding the wider confidence intervals of the effect measures resulting from the reduced sample size (Table 4, right).

The overall picture was substantially unchanged in the unmatched sensitivity analyses (ULR-1 and ULR-2), even though there was a progressive attenuation of the measures of association as the size of the dataset increased, independently of the exposure metric (Table 5). Corresponding analyses restricted to ALL provided a similar pattern of results (Table S3).

**Table 5:** ORs for childhood leukemia according to ELF-MF exposure (ULR adjusted *).

		ULR-1				ULR-2			
Exposure Metric	Level (μT)	Cases	Controls	OR	95% CI	Cases	Controls	OR	95% CI
AM continuous	per 1 μT increase	519	784	0.76	0.25–2.32	537	825	0.68	0.22–2.09
AM categorical	≤0.1	468	721	1.00	-	482	753	1.00	-
	(0.1–0.2]	37	38	1.58	0.98–2.53	41	43	1.55	0.99–2.44
	>0.2	14	25	0.88	0.45–1.72	14	29	0.77	0.40–1.48
GM categorical	≤0.1	478	732	1.00	-	494	765	1.00	-
	(0.1–0.2]	33	34	1.57	0.95–2.59	35	39	1.46	0.91–2.36
	>0.2	8	18	0.68	0.29–1.59	8	21	0.60	0.26–1.37
P95 (3 levels)	≤0.1	423	633	1.00	-	435	659	1.00	-
	(0.1–0.2]	52	95	0.83	0.57–1.19	54	101	0.81	0.57–1.16
	>0.2	44	56	1.21	0.80–1.84	48	65	1.15	0.77–1.72
P95 (4 levels)	≤0.1	423	633	1.00	-	435	659	1.00	-
	(0.1–0.2]	52	95	0.83	0.57–1.19	54	101	0.82	0.57–1.16
	(0.2–0.3]	24	22	1.76	0.97–3.22	28	25	1.81	1.03–3.18
	>0.3	20	34	0.87	0.49–1.55	20	40	0.76	0.43–1.32

ULR adjusted * = unconditional logistic regression models, adjusted for age, sex, region, and parents’ educational level; ULR-1 = first series of unconditional logistic regression analyses including matched and unmatched cases and controls from sets originally strictly matched on age; ULR-2 = second series of unconditional logistic regression analyses including matched and unmatched cases and controls, independent of compliance with the strict age-matching criterion; AM = arithmetic mean of instantaneous values from 24 to 48 h bedroom measurements; GM = geometric mean of instantaneous values from 24 to 48 h bedroom measurements; P95 = 95th percentile of instantaneous values from 24 to 48 h bedroom measurements.

Findings from CLR analyses based on nighttime (10 pm to 5:59 am) exposure metrics are outlined in Table 6 (all leukemias on the left, ALL on the right). Compared to findings presented in Table 4, reduced ORs for all leukemias in relation to most nighttime exposure metrics (continuous and categorical AM, categorical GM, P95 at three and four levels) were observed (Table 6, left). The trend was similar in the nighttime CLR analyses including only ALL cases and controls (Table 6, right), with few exceptions (increased ORs in the analyses based on the categorical GM exposure variable).

The unmatched sensitivity analyses (ULR-1 and ULR-2) based on nighttime exposure metrics showed an overall pattern of findings comparable to that observed in the matched analyses, apart from a generalized progressive attenuation of all measures of association with increasing sample size (Table S4—All leukemias; Table S5—ALL).

The analyses aimed at exploring possible variations in findings resulting from different methods of non-detect treatment (carried out on the ULR-2 dataset, all leukemias) showed that the arithmetic mean is less sensitive than the geometric mean to changes in the correction factor (Table S6).

In the sensitivity analyses carried out on the ULR-2 (all leukemias) dataset, the exclusion of 6 cases with Down syndrome (Table S7) did not imply any appreciable variation in findings compared to those described in Table 5—right side.

**Table 6:** ORs for childhood leukemia according to nighttime ^§^ ELF-MF exposure (CLR—Adjusted *).

		All Leukemias				Acute Lymphocytic Leukemia			
Exposure Metric	Level (μT)	Cases	Controls	OR	95% CI	Cases	Controls	OR	95% CI
AM continuous	per 1 μT increase	409	569	0.62	0.13–2.90	356	499	0.79	0.15–4.11
AM categorical	≤0.1	375	531	1.00	-	326	466	1.00	-
	(0.1–0.2]	26	24	1.57	0.85–2.90	22	20	1.54	0.80–2.96
	>0.2	8	14	0.67	0.27–1.68	8	13	0.74	0.29–1.89
GM categorical	≤0.1	377	534	1.00	-	327	468	1.00	-
	(0.1–0.2]	28	21	1.87	1.01–3.45	25	18	1.87	0.98–3.56
	>0.2	4	14	0.32	0.10–1.00	4	13	0.36	0.11–1.13
P95 (3 levels)	≤0.1	352	493	1.00		307	433	1.00	-
	(0.1–0.2]	37	46	1.12	0.68–1.83	31	41	1.01	0.59–1.72
	>0.2	20	30	0.84	0.45–1.54	18	25	0.92	0.48–1.77
P95 (4 levels)	≤0.1	352	493	1.00	-				
	(0.1–0.2]	37	46	1.11	0.67–1.82				
	(0.2–0.3]	15	11	2.29	0.95–5.50				
	>0.3	5	19	0.28	0.10–0.77				

^§^ Nighttime = from 10 pm to 5:59 am; * CLR—Adjusted= conditional logistic regression models, adjusted for parents’ education; AM = arithmetic mean of instantaneous values from nighttime bedroom measurements; GM = geometric mean of instantaneous values from nighttime bedroom measurements; P95 = 95th percentile of instantaneous values from nighttime bedroom measurements.

The results of analyses further restricted to subjects living at the time of measurements in homes inhabited since conception and/or birth (Table 7) did not differ from those based on the whole ULR-2 dataset.

**Table 7:** Sensitivity analyses—Further restriction on the house: ORs for childhood leukemia (all types), according to ELF-MF exposure (arithmetic mean of 24–48 h bedroom measurements) in the subset of children still living in the dwelling inhabited since conception and/or birth (unconditional logistic regression models ULR-2, adjusted for age, sex, region, and parents’ educational level).

Residency in the Home	Level (μT)	Cases	Controls	OR	95% CI	
Since conception	≤0.1	344	574	1.00		
	(0.1–0.2]	28	31	1.57	0.91	2.70
	>0.2	12	22	0.91	0.44	1.89
Since birth	≤0.1	371	604	1.00		
	(0.1–0.2]	31	34	1.53	0.92	2.57
	>0.2	12	25	0.78	0.38	1.60
Since conception and birth	≤0.1	343	571	1.00		
	(0.1–0.2]	28	31	1.57	0.91	2.70
	>0.2	12	22	0.91	0.44	1.91

Similarly negligible impact had the application of stricter criteria for inclusion in the analyses, such as to measurements lasting ≥48 h (Table S8), with sampling interval of 30 s (Table S9), or made during the week-end (Table S10).

## 4. Discussion and Conclusions

The nationwide SETIL case-control provided the first estimates of ELF-MF exposure among Italian children. The average level of magnetic field induction measured in the child’s bedroom (24–48 h TWA, geometric mean) was 0.04 μT, and less than 2% of subjects was exposed above 0.3 μT (0.6% cases, 1.6% controls), in line with available estimates for European children [17].

The proportions of participant control families (71% interviewed, 61% accepted ELF-MF bedroom measurements) were comparable to those observed in previous studies with similar design [2,5].

As to the relationship between childhood leukemia and exposure to ELF-MF, no association was observed in the current study in the analyses based on the continuous arithmetic mean as exposure variable, no matter of the statistical approach (CLR or ULR models). Findings from the analyses based on categorical exposure variables were characterized by incoherent exposure-outcome relationships, whereas increased ORs (between 1.5 and 2, of borderline statistical significance) were often observed at low exposure levels, along with markedly reduced ORs at the highest exposure level, even in the opposite direction (*i.e.*, below 1). Variations in the exposure metric used implied little changes in the pattern of findings from the categorical analyses. Compared to the categorical arithmetic mean, the exposure classification based on the geometric mean resulted in a less accentuated tendency for the ORs at the lower and upper exposure categories to diverge (*i.e.*, to depart from the null value in different directions), while the opposite occurred when employing the P95 (4-level categorization).

Thus, our results seem not in line with the available epidemiological evidence [3,4,5,7,18,19]. Rather, there is a certain similarity between our findings and those related to the analyses of childhood leukemia and indoor magnetic field levels within the more recently published Northern California Childhood Leukemia Study [20].

Observational epidemiological studies are inherently susceptible to bias from multiple sources [21]. For this reason, in the framework of etiological research, results provided by any single epidemiological study are often (if not always) difficult to interpret, and our findings are no exception to the rule.

The main drawbacks of our study are the low power at moderate/high levels of estimated exposure, along with susceptibility to participation bias, exposure assessment errors, and confounding. Due to the overall low level of ELF-MF exposure in our study population, few cases and controls were classified in the upper exposure categories considered in previous studies. Thus, the incoherent findings from the categorical analyses may be due to random variation. However, an undetected source of bias may also be at play. In fact, the confidence interval of the ORs at the highest exposure category (>0.2 µT for the arithmetic or geometric means; >0.3 µT for P95) often did not include the point estimate of the OR at the lowest exposure level. This suggests that distortion is a more likely explanation for these findings than the low power of our study at the highest exposure levels.

The potential for selection bias is high in our study, as it was in most previous studies on the relationship between childhood leukemia and ELF-MF exposure [22,23,24,25,26]. Participation rates were lower among controls than cases, and participation was also associated with educational level of parents. The tendency observed in the unmatched ULR analyses (compared to findings from the CLR models) for the ORs at the intermediate and high exposure levels to regress towards the null value, might be explained by varying degree of selection bias in datasets of different size; that is, the impact of a distortion originating from a participation associated with both the exposure and the disease could be stronger in the smallest CLR dataset (restricted to children complying with the strictest inclusion criteria), than in the increasingly larger datasets of subjects included in the ULR-1, and ULR-2 analyses.

We do acknowledge that a participation bias whose amount and direction varies depending on the level of estimated exposure may be difficult to conceive. However, there is some empirical evidence in support of this hypothesis. In the context of the SETIL study, we carried out a small pilot study of childhood leukaemia and exposure to benzene assessed by repeated seasonal weekly measurements in breathing zone air samples and outside the children’s dwellings [27]. In this side study, we had the opportunity to estimate the amount and direction of participation bias, as well as the correlation between average yearly outdoor benzene concentrations and 24–48 h ELF-MF level in the child’s bedroom, taking into account participation rates [27]. Exposures to benzene and ELF-MF were positively correlated, and the strength of the association was greater among full-participants in the personal benzene monitoring, than among subjects with available outdoor benzene levels only. Benzene concentrations in proximity of the subjects’ homes were lower among participant controls compared to non-participants, but did not differ between participant and non-participant cases; the direction of the participation bias was found to depend on the cut-point chosen to distinguish exposed and unexposed, with no bias when benzene exposure was categorized around the median (3.25 μg/m^3^; bias factor = 1.03), and biases in the opposite directions when cut-offs at P75 (4.34 μg/m^3^) and at 5 μg/m^3^ were used (bias factors = 0.64 and 1.42, respectively) [27].

The difficulty of reliably assessing exposure in the years preceding diagnosis is a common problem in all case-control studies. Concerning ELF-MF, it is not clear how well contemporaneous measurements accurately characterize personal exposure in general, and especially during the etiological time window [7,28]. Restriction of eligibility for the ELF-MF survey to children still living at the time of the interview and measurements in the home inhabited one year before the diagnosis/reference date, applied in the current study, was aimed at minimizing this information bias (unavoidable, at any rate, in retrospective study designs). A further reduction of the same bias was the aim of restricting the analyses to subjects living at the time of measurements in homes inhabited since conception and/or birth.

In a pilot study including a random sample of 113 dwellings from 5 Italian regions, we evaluated the representativeness of 24–48 h measurements of ELF-MF in the child’s bedroom, compared to a measurement duration of 5 days; the analysis of the 24-h moving averages showed that the 5-day measurement close to the child’s bed could be shortened to 24–48 h without appreciable effects on the estimated mean magnetic field; moreover, there was no difference between the mean magnetic field measured in weekends *vs.* workdays [13].

The original measurement protocol prescribed the use of EMDEX II^®^ meters for short-term measurements and of EMDEX Lite^®^ meters for long-term measurements. In practice, this clause could not be maintained because of the temporary unavailability of exposure meters. Long-term measurements were therefore conducted using either type of meters. The possible implications of this deviation from the protocol were assessed in a reliability and agreement side-study. In a calibration setting it was found that the EMDEX Lite^®^ exhibited a dependence of measurement error on the orientation of the instrument in the magnetic field, whereas the EMDEX II^®^ did not [29]. This finding is, however, unlikely to play any role in a real setting, as the orientation of the instrument in the magnetic field to be measured is likely to be random. Actually, adjustment for type of instrument in the analysis did not evidence any confounding effect.

To address a possible underestimation of risk due to random exposure misclassification, we carried out analyses based on nighttime exposure metrics. The underlying rationale is the following. Assuming a non-null exposure-outcome relationship, if the amount of random exposure error were indeed lower for the nighttime exposure metrics compared to the metrics based on complete 24–48 h measurements, both an overall increase in the ORs among the exposed, and a positive trend in risk estimates across increasing exposure levels, would be expected in such analyses. Contrary to expectation, however, findings from the analyses based on nighttime exposure metrics did not differ from results obtained using exposure metrics from the entire 24–48 h recordings; this observation, consistent with previous studies [30], does not support the hypothesis that nighttime measures are more accurate exposure proxies, and detracts from attributing our findings to random exposure misclassification. Moreover, an exposure measurement error whose amount and direction varied depending on the exposure level (*i.e.*, increasing with increasing exposure level, so as to mask or invert an exposure-outcome trend), would be needed for information bias to be able to explain our findings, which is difficult to figure out.

Confounding, due to correlated causes of the disease under study, may bias the empirical measures of association in either direction (toward or away from the null). For this reason, confounding is an issue in the interpretation of any epidemiological study, irrespective of its finding (*i.e.*, positive, negative, or null exposure-outcome associations) [31,32]. However, none of the many potentially confounding variables assessed in the current study implied changes in the effect estimates greater than 10%. Moreover, traffic-related air pollution (estimated using various exposure assessment strategies), benzene (assessed by repeated personal measurements), and smoking habits of the parents, appeared not to affect the incidence of childhood leukaemia in the SETIL study [27,33,34]. On the other hand, a previous analysis of the SETIL study reported associations between childhood leukemia risk and parental occupational exposure to some chemical agents (maternal exposure to aliphatic and aromatic hydrocarbons, and paternal exposure to diesel exhaust, lead and mineral oil) [35]. We did not formally assess whether preconceptional occupational exposures of parents to chemicals confounded the relationship between CL and ELF-MF exposure; however, due to the small number of cases and controls underlying those findings, it is very unlikely that they can explain the current results.

In summary, the SETIL study provides a limited contribution to the epidemiological evidence concerning the relation between childhood leukemia and exposure to ELF-MF. Due to the low prevalence of above background exposure levels, our findings are noninformative on the relative risk of disease among the exposed above 0.3 μT. The incoherent exposure-outcome relationships observed might be due to distortions from multiple sources, with an unusual association with the true exposure level.

As suggested by other authors, after three decades of epidemiologic investigation on the relationship of ELF-MF to childhood leukemia, little can be gained from further repetition of investigations of risks at moderate and low exposure levels, unless such studies can be designed to test specific hypotheses, such as selection bias, aspects of exposure not previously captured, co-exposure to other chemical or physical agents, or gene-environment interactions [6,7]. That notwithstanding, our study may contribute to future meta- or pooled analyses. Moreover, the SETIL study provides information on intensity and variability of ELF-MF exposure in quite a large sample of population controls from all over Italy, which represent parameters required to calculate estimates of attributable risks, and for this reason it may be considered a valuable contribution in the public health and risk communication perspectives.

## Supplementary Files

Supplementary File 1
