# Supplementary material for: Childhood Leukemia and 50 Hz Magnetic Fields: Findings from the Italian SETIL Case-Control Study
Source: Int J Environ Res Public Health. 2015 Feb 16;12(2):2184–204. doi: 10.3390/ijerph120202184 (PMC4344719; doi:10.3390/ijerph120202184)
Supplement: Supplementary File 1 [file ijerph-12-02184-s001.pdf]

Childhood Leukemia and 50 Hz Magnetic Fields: Findings from the Italian SETIL Case-Control Study

Table S1. Descriptive characteristics of cases and controls included in the unconditional logistic regression models analyses.

	Cases				Controls			
	ULR-1		ULR-2		ULR-1		ULR-2	
	N°	%	N°	%	N°	%	N°	%
**Sex**								
Male	281	53.8	290	53.7	426	54.0	447	53.9
Female	241	46.2	250	46.3	363	46.0	383	46.1
**Age at diagnosis years**								
[0, 2)	71	13.6	73	13.5	121	15.3	131	15.8
[2, 4)	189	36.2	196	36.3	263	33.3	272	32.8
[4, 6)	115	22.0	119	22.0	170	21.6	180	21.7
[6, 10]	147	28.2	152	28.2	235	29.8	247	29.8
**Father's education**								
Primary school (8 years)	255	48.8	269	49.8	332	42.1	359	43.3
High school (12-13 years)	212	40.6	216	40.0	331	42.0	341	41.1
University (≥15 years)	52	10.0	52	9.6	121	15.3	125	15.1
Missing	3	0.57	3	0.56	5	0.63	5	0.60
**Mother's education**								
Primary school (8 years)	238	45.6	252	46.7	291	36.9	311	37.5
High school (12-13 years)	226	43.3	230	42.6	386	48.9	405	48.8
University (≥15 years)	58	11.1	58	10.7	112	14.2	114	13.7
Missing	0	-	0	-	0	-	0	-
**Children in the family**								
1	144	27.6	149	27.6	209	26.5	228	27.5
2	279	53.4	288	53.3	436	55.3	453	54.6
3+	99	19.0	103	19.1	144	18.2	149	17.9
**Mother's smoking (during pregnancy)**								
Yes	58	11.1	59	10.9	87	11.0	90	10.8
No	464	88.9	481	89.1	701	88.9	738	88.9
Missing	0	-	0	-	1	0.13	2	0.24
**Child's exposure to second-hand smoke (at diagnosis)**								
Yes	160	30.6	168	31.1	237	30.0	253	30.5
No	360	69.0	370	68.5	547	69.3	571	68.8
Missing	2	0.38	2	0.37	5	0.63	6	0.72

Table S1. *Cont.*

	Cases				Controls			
	ULR-1		ULR-2		ULR-1		ULR-2	
	N°	%	N°	%	N°	%	N°	%
**Type of home (at interview and measurements)**								
Detached house	127	24.3	134	24.8	208	26.4	225	27.1
Apartment building	388	74.3	399	73.9	573	72.6	597	71.9
Missing	7	1.3	7	1.3	8	1.0	8	0.96

ULR-1 = first series of unconditional logistic regression analyses including matched and unmatched cases and controls from sets originally strictly matched on age; ULR-2 = second series of unconditional logistic regression analyses including matched and unmatched cases and controls, independent of compliance with the strict age-matching criterion.

Table S2. ELF-MF level (μT) in the child's bedroom of cases and controls with valid measurements, according to different exposure metrics and methods of non-detect correction.

		Cases (# 540)				Controls (# 830)			
**Metric**	**Correction**	**Mean**	**SD**	**Min**	**Max**	**Mean**	**SD**	**Min**	**Max**
**AM**	None	0.0401	0.0865	0	1.609	0.0449	0.1208	0	2.524
	0.005	0.0412	0.0861	0.005	1.609	0.0459	0.1205	0.005	2.524
	0.007	0.0416	0.0860	0.007	1.609	0.0463	0.1204	0.007	2.524
**GM**	0.0001	0.0333	0.0789	0.0001	1.485	0.0375	0.1108	0.0001	2.499
	0.005	0.0356	0.0782	0.005	1.485	0.0395	0.1102	0.005	2.499
	0.007	0.0362	0.0780	0.007	1.485	0.0400	0.1101	0.007	2.499
**P90**	None	0.0621	0.1329	0	2.380	0.0694	0.1564	0	2.730
	0.0001	0.0622	0.1329	0.0001	2.380	0.0695	0.1564	0.0001	2.730
	0.005	0.0625	0.1327	0.005	2.380	0.0698	0.1563	0.005	2.730
	0.007	0.0627	0.1326	0.007	2.380	0.0699	0.1562	0.007	2.730
**P95**	None	0.0737	0.1585	0	2.910	0.0788	0.1651	0	2.810
	0.0001	0.0738	0.1585	0.0001	2.910	0.0789	0.1651	0.0001	2.810
	0.005	0.0740	0.1584	0.005	2.910	0.0790	0.1650	0.005	2.810
	0.007	0.0741	0.1583	0.007	2.910	0.0791	0.1650	0.007	2.810
**P99**	None	0.1186	0.3445	0	6.360	0.1099	0.2080	0	2.880
	0.0001	0.1187	0.3445	0.0001	6.360	0.1100	0.2080	0.0001	2.880
	0.005	0.1187	0.3444	0.005	6.360	0.1100	0.2079	0.005	2.880
	0.007	0.1187	0.3444	0.007	6.360	0.1100	0.2079	0.007	2.880

Valid measurements = made in homes inhabited one year before reference date & duration ≥24 h; Correction = value assigned to instantaneous measurements below the detection limit (0.01 μT); AM = arithmetic mean of instantaneous values from 24 to 48 h bedroom measurements; GM = geometric mean of instantaneous values from 24 to 48 h bedroom measurements; P90, P95, P99 = 90th, 95th and 99th percentiles of instantaneous values from 24 to 48 h bedroom measurements.

Table S3. Sensitivity analyses: ORs for acute lymphocytic leukemia (ALL) according to ELF-MF exposure (unconditional logistic regression models, adjusted for age, sex, region, and parents' educational level).

		ULR-1					ULR-2				
**Exposure Metric**	**Level (μT)**	**Cases**	**Controls**	**OR**	**95% CI**		**Cases**	**Controls**	**OR**	**95% CI**	
AM continuous	per 1 μT increase	453	680	0.98	0.23	4.15	470	719	0.79	0.19	3.22
AM categorical	≤0.1	410	626	1.00			423	656	1.00		
	(0.1–0.2]	30	32	1.50	0.89	2.51	34	37	1.49	0.91	2.43
	>0.2	13	22	0.93	0.46	1.90	13	26	0.81	0.41	1.60
GM categorical	≤0.1	419	635	1.00			434	666	1.00		
	(0.1–0.2]	27	29	1.48	0.86	2.56	29	34	1.38	0.82	2.32
	>0.2	7	16	0.68	0.27	1.68	7	19	0.59	0.24	1.42
P95 (3 levels)	≤0.1	373	547	1.00			385	572	1.00		
	(0.1–0.2]	42	86	0.72	0.48	1.07	43	92	0.69	0.47	1.03
	>0.2	38	47	1.25	0.79	1.96	42	55	1.19	0.78	1.83

ULR-1 = first series of unconditional logistic regression analyses including matched and unmatched cases and controls from sets originally strictly matched on age; ULR-2 = second series of unconditional logistic regression analyses including matched and unmatched cases and controls, independent of compliance with the strict age-matching criterion; AM = arithmetic mean of instantaneous values from individual 24 to 48 h measurements; GM = geometric mean of instantaneous values from individual 24 to 48 h measurements; P95 = 95th percentile of the distribution of values from individual 24 to 48 h measurements.

Table S4. Sensitivity analyses: ORs for childhood leukemia (all types), according to nighttime * ELF-MF exposure (unconditional logistic regression models, adjusted for age, sex, region, and parents' educational level).

		ULR-1					ULR-2				
**Exposure Metric**	**Level (μT)**	**Cases**	**Controls**	**OR**	**95% CI**		**Cases**	**Controls**	**OR**	**95% CI**	
AM continuous	per 1 μT increase	518	784	0.60	0.19	1.82	536	825	0.54	0.17	1.68
AM categorical	≤0.1	475	727	1.00			492	760	1.00		
	(0.1–0.2]	31	35	1.41	0.85	2.33	32	40	1.28	0.79	2.08
	>0.2	12	22	0.82	0.40	1.68	12	25	0.73	0.36	1.49
GM categorical	≤0.1	478	732	1.00			495	766	1.00		
	(0.1–0.2]	33	31	1.65	0.99	2.75	34	35	1.52	0.93	2.49
	>0.2	7	21	0.50	0.21	1.20	7	24	0.45	0.19	1.07
P95 (3 levels)	≤0.1	448	673	1.00			461	703	1.00		
	(0.1–0.2]	45	66	1.07	0.72	1.61	50	71	1.11	0.75	1.64
	>0.2	25	45	0.82	0.49	1.37	25	51	0.74	0.45	1.23
P95 (4 levels)	<=0.1	448	673	1.00			461	703	1.00		
	(0.1–0.2]	45	66	1.07	0.72	1.61	50	71	1.11	0.75	1.64
	(0.2–0.3]	16	19	1.27	0.64	2.51	16	21	1.18	0.60	2.30
	>0.3	9	26	0.51	0.23	1.10	9	30	0.45	0.21	0.96

Nighttime = 10 pm–5:59 am; ULR-1 = first series of unconditional logistic regression analyses including matched and unmatched cases and controls from sets originally strictly matched on age; ULR-2 = second series of unconditional logistic regression analyses including matched and unmatched cases and controls, independent of compliance with the strict age-matching criterion; AM = arithmetic mean of instantaneous values from individual 24 to 48 h measurements; GM = geometric mean of instantaneous values from individual 24 to 48 h measurements; P95 = 95th percentile of the distribution of values from individual 24 to 48 h measurements.

Table S5. Sensitivity analyses: ORs for childhood acute lymphocytic leukemia (ALL) according to nighttime * ELF-MF exposure (unconditional logistic regression models, adjusted for age, sex, region, and parents' educational level).

		**ULR-1**					**ULR-2**				
Exposure metric	Level (μT)	Cases	Controls	OR	95% CI		Cases	Controls	OR	95% CI	
AM continuous	per 1 μT increase	452	680	0.68	0.18	2.57	469	719	0.59	0.16	2.20
AM categorical	≤0.1	415	630	1.00			431	661	1.00		
	(0.1–0.2]	26	30	1.37	0.79	2.37	27	35	1.24	0.73	2.09
	>0.2	11	20	0.82	0.39	1.75	11	23	0.73	0.35	1.53
GM categorical	≤0.1	416	634	1.00			432	666	1.00		
	(0.1–0.2]	29	27	1.66	0.96	2.86	30	31	1.51	0.90	2.55
	>0.2	7	19	0.56	0.23	1.35	7	22	0.50	0.21	1.18
P95 (3 levels)	≤0.1	393	582	1.00			405	610	1.00		
	(0.1–0.2]	37	61	0.93	0.60	1.44	42	66	0.99	0.65	1.49
	>0.2	22	37	0.88	0.51	1.53	22	43	0.78	0.46	1.34

Nighttime = 10 pm–5:59 am; ULR-1 = first series of unconditional logistic regression analyses including matched and unmatched cases and controls from sets originally strictly matched on age; ULR-2 = second series of unconditional logistic regression analyses including matched and unmatched cases and controls, independent of compliance with the strict age-matching criterion; AM = arithmetic mean of instantaneous values from individual 24 to 48 h measurements; GM = geometric mean of instantaneous values from individual 24 to 48 h measurements; P95 = 95th percentile of the distribution of values from individual 24 to 48 h measurements.

Table S6. Sensitivity analyses—method of non-detect correction: ORs for childhood leukemia (all types), according to ELF-MF exposure (unconditional logistic regression models, adjusted for age, sex, region, and parents' educational level).

Exposure Metric	Correction	Level (µT)	Cases	Controls	OR	95% CI	
AM	None	≤0.1	482	753	1.00		
		(0.1–0.2]	41	43	1.55	0.99	2.44
		>0.2	14	29	0.77	0.40	1.48
AM	0.005	≤0.1	482	753	1.00		
		(0.1–0.2]	41	43	1.55	0.99	2.44
		>0.2	14	29	0.77	0.40	1.48
GM	0.0001	≤0.1	494	765	1.00		
		(0.1–0.2]	35	39	1.46	0.91	2.36
		>0.2	8	21	0.60	0.26	2.36
GM	0.005	≤0.1	493	765	1.00		
		(0.1–0.2]	36	39	1.51	0.94	2.42
		>0.2	8	21	0.60	0.26	1.37
P95	None	≤0.1	435	659	1.00		
		(0.1–0.2]	54	101	0.81	0.57	1.16
		>0.2	48	65	1.15	0.77	1.72
P95	0.0001	≤0.1	426	643	1.00		
		(0.1–0.2]	58	111	0.78	0.55	1.10
		>0.2	53	71	1.16	0.79	1.70
P95	0.005	≤0.1	435	659	1.00		
		(0.1–0.2]	54	101	0.81	0.57	1.16
		>0.2	48	65	1.15	0.77	1.72

Correction = value assigned to instantaneous measurements below the detection limit (0.01 µT); AM = arithmetic mean of instantaneous values from individual 24 to 48 h measurements; GM = geometric mean of instantaneous values from individual 24 to 48 h measurements; P95 = 95th percentile of the distribution of values from individual 24 to 48 h measurements.

Table S7. Sensitivity analyses—exclusion of six cases with Down syndrome: ORs for childhood leukemia (all types) according to ELF-MF exposure (unconditional logistic regression models, adjusted for age, sex, region, and parents' educational level).

Exposure Metric	Level (µT)	Cases	Controls	OR	95% CI	
AM continuous	per 1 µT increase	534	830	0.68	0.22	2.11
AM categorical	≤0.1	477	753	1	-	-
	(0.1–0.2]	40	43	1.53	0.97	2.41
	>0.2	14	29	0.78	0.41	1.51
GM categorical	≤0.1	489	756	1	-	-
	(0.1–0.2]	34	39	1.44	0.89	2.33
	>0.2	8	21	0.60	0.26	1.39

Table S7. *Cont.*

Exposure Metric	Level (µT)	Cases	Controls	OR	95% CI	
P95 (3 levels)	≤0.1	431	659	1	-	-
	(0.1–0.2]	54	101	0.82	0.58	1.176
	>0.2	46	65	1.11	0.74	1.67
P95 (4 levels)	≤0.1	431	659	1	-	-
	(0.1–0.2]	54	101	0.83	0.58	1.18
	(0.2–0.3]	27	25	1.77	1.00	3.12
	>0.3	19	40	0.73	0.41	1.28

AM = arithmetic mean of instantaneous values from individual 24 to 48 h measurements; GM = geometric mean of instantaneous values from individual 24 to 48 h measurements; P95 = 95th percentile of the distribution of values from individual 24 to 48 h measurements.

Table S8. Sensitivity analyses—further restriction to measurements lasting ≥48 h: ORs for childhood leukemia (all types) according to ELF-MF exposure (unconditional logistic regression models, adjusted for age, sex, region, and parents' educational level).

Exposure Metric	Level (µT)	Cases	Controls	OR	95% CI	
AM continuous	per 1 µT increase	431	652	0.63	0.19	2.13
AM categorical	≤0.1	394	603	1.00		
	(0.1–0.2]	29	30	1.56	0.91	2.66
	>0.2	8	19	0.64	0.27	1.49
GM categorical	≤0.1	403	608	1.00		
	(0.1–0.2]	24	30	1.26	0.72	2.21
	>0.2	4	14	0.43	0.14	1.34
P95 (3 levels)	≤0.1	355	531	1.00		
	(0.1–0.2]	43	74	0.90	0.60	1.34
	>0.2	33	47	1.07	0.67	1.72
P95 (4 levels)	≤0.1	355	531	1.00		
	(0.1–0.2]	43	74	0.90	0.60	1.35
	(0.2–0.3]	18	19	1.52	0.78	2.96
	>0.3	15	28	0.78	0.41	1.50

AM = arithmetic mean of instantaneous values from individual 24-48 h measurements; GM = geometric mean of instantaneous values from individual 24 to 48 h measurements; P95 = 95th percentile of the distribution of values from individual 24 to 48 h measurements.

Table S9. Sensitivity analyses—further restriction to measurements with sampling interval of 30 s: ORs for childhood leukemia (all types) according to ELF-MF exposure (unconditional logistic regression models, adjusted for age, sex, region, and parents' educational level).

Exposure Metric	Level (μT)	Cases	Controls	OR	95% CI	
AM continuous	per 1 μT increase	531	822	0.66	0.21	2.05
AM categorical	≤0.1	477	750	1.00		
	(0.1–0.2]	41	43	1.56	0.99	2.45
	>0.2	13	29	0.72	0.37	1.41
GM categorical	≤0.1	489	762	1.00		
	(0.1–0.2]	34	39	1.43	0.88	2.31
	>0.2	8	21	0.60	0.26	1.37
P95 (3 levels)	≤0.1	430	656	1.00		
	(0.1–0.2]	53	101	0.80	0.56	1.15
	>0.2	48	65	1.16	0.78	1.72
P95 (4 levels)	≤0.1	430	656	1.00		
	(0.1–0.2]	53	101	0.81	0.56	1.15
	(0.2–0.3]	28	25	1.82	1.03	3.19
	>0.3	20	40	0.76	0.44	1.33

AM = arithmetic mean of instantaneous values from individual 24 to 48 h measurements; GM = geometric mean of instantaneous values from individual 24 to 48 h measurements; P95 = 95th percentile of the distribution of values from individual 24 to 48 h measurements.

Table S10. Sensitivity analyses—further restriction to measurements made during the week-end: ORs for childhood leukemia (all types) according to ELF-MF exposure (unconditional logistic regression models, adjusted for age, sex, region, and parents' educational level).

Exposure Metric	Level (μT)	Cases	Controls	OR	95% CI	
AM continuous	per 1 μT increase	309	450	0.94	0.27	3.27
AM categorical	≤0.1	279	415	1.00		
	(0.1–0.2]	21	23	1.42	0.76	2.66
	>0.2	9	12	1.14	0.47	2.78
GM categorical	≤0.1	280	418	1.00		
	(0.1–0.2]	25	22	1.78	0.97	3.26
	>0.2	4	10	0.64	0.19	2.09
P95 (3 levels)	≤0.1	259	367	1.00		
	(0.1–0.2]	25	45	0.78	0.46	1.32
	>0.2	25	38	0.95	0.55	1.64

Table S10. *Cont.*

Exposure Metric	Level (µT)	Cases	Controls	OR	95% CI	
P95 (4 levels)	<=0.1	259	367	1.00		
	(0.1–0.2]	25	45	0.79	0.47	1.33
	(0.2–0.3]	16	15	1.61	0.76	3.39
	>0.3	9	23	0.56	0.25	1.23

AM = arithmetic mean of instantaneous values from individual 24 to 48 h measurements; GM = geometric mean of instantaneous values from individual 24 to 48 h measurements; P95 = 95th percentile of the distribution of values from individual 24 to 48 h measurements.

© 2015 by the authors; licensee MDPI, Basel, Switzerland. This article is an open access article distributed under the terms and conditions of the Creative Commons Attribution license (http://creativecommons.org/licenses/by/4.0/).
